# Economic Value of Lost Productivity Attributable to Human Papillomavirus Cancer Mortality in the United States

**DOI:** 10.3389/fpubh.2020.624092

**Published:** 2021-02-16

**Authors:** Masoom Priyadarshini, Vimalanand S. Prabhu, Sonya J. Snedecor, Shelby Corman, Barbara J. Kuter, Chizoba Nwankwo, Diana Chirovsky, Evan Myers

**Affiliations:** ^1^Pharmerit – an OPEN Health Company, Bethesda, MD, United States; ^2^Merck & Co. Inc., Kenilworth, NJ, United States; ^3^Division of Women's Community and Population Health, Department of Obstetrics & Gynecology, Duke University School of Medicine, Durham, NC, United States

**Keywords:** human papillomavirus, productivity loss, mortality, cervical cancer, oropharyngeal cancer

## Abstract

**Objectives:** To estimate years of potential life lost (YPLL) and present value of future lost productivity (PVFLP) associated with premature mortality due to HPV-attributable cancers, specifically those targeted by nonavalent HPV (9vHPV) vaccination, in the United States (US) before vaccine use.

**Methods:** YPLL was estimated from the reported number of deaths in 2017 due to HPV-related cancers, the proportion attributable to 9vHPV-targeted types, and age- and sex-specific US life expectancy. PVFLP was estimated as the product of YPLL by age- and sex-specific probability of labor force participation, annual wage, value of non-market labor, and fringe benefits markup factor.

**Results:** An estimated 7,085 HPV-attributable cancer deaths occurred in 2017 accounting for 154,954 YPLL, with 6,482 deaths (91%) and 141,019 YPLL (91%) attributable to 9vHPV-targeted types. The estimated PVFLP was $3.8 billion for cancer deaths attributable to 9vHPV-targeted types (84% from women). The highest productivity burden was associated with cervical cancer in women and anal and oropharyngeal cancers in men.

**Conclusions:** HPV-attributable cancer deaths are associated with a substantial economic burden in the US, much of which could be vaccine preventable.

## Introduction

Human papillomavirus (HPV) can cause cervical, vulvar, vaginal, penile, anal, and oropharyngeal cancers (1). The median age at diagnosis of HPV-associated cancer in the United States (US) ranges from 49 years for cervical cancer to 69 years for penile cancer, with most diagnoses across other HPV-associated cancer types occurring in patients in their early- to mid-60s (2). Between 2012 and 2016, ~44,000 HPV-associated cancers (i.e., cases in sites where HPV is often found) were reported per year. Of these, an estimated 34,800 annual cases (79%) were attributable to HPV (defined by the US Centers for Disease Control and Prevention [CDC] as cancers that are “probably caused” by HPV) (3).

The quadrivalent HPV vaccine (4vHPV), which targets high-risk HPV 16 and 18 as well as low-risk HPV 6 and 11, was introduced in the US in 2006 and was the predominant vaccine administered through 2015 (4). The nonavalent HPV vaccine (9vHPV), which targets HPV 31, 33, 45, 52, and 58 in addition to the 4vHPV-targeted types, was licensed in late 2014 and since late 2016 has been the only HPV vaccine sold in the US (4). Although HPV vaccination coverage in the US has improved over the past decade, many adolescents and young adults remain unvaccinated (5, 6). In the 2019 National Immunization Survey-Teen (NIS-Teen), 54.2% of adolescents aged 13 to 17 years were up to date with the HPV vaccine series, and 71.5% had received at least one dose of HPV vaccine (7). However, coverage as estimated by the National Health and Nutrition Examination Survey (NHANES) and National Health Interview Survey (NHIS) is much lower in adolescents and adults (5, 8).

An estimated 32,100 HPV-attributable cancers (92%) per year are attributable to HPV 16, 18, 31, 33, 45, 52, and 58, the high-risk types targeted by the 9vHPV vaccine (1, 3). Studies of data collected during the 4vHPV era (i.e., 2006–2014) demonstrated a declining prevalence of HPV 16/18 in young women and young men in the US (9, 10), as well as a reduced prevalence of cervical precancer (4, 11, 12) and statistically significant reductions in cervical cancer incidence among young women in the US (13). More definitive data for HPV-associated cancers are likely to become available in the next few years. In time, HPV vaccination may help reduce the incidence of HPV-attributable cancer and thereby reduce premature mortality and morbidity due to HPV-attributable cancers (1).

Cancer-related mortality has a wide impact on society and individuals, including the grief of bereaved family members and friends (14, 15). Economists use productivity loss—i.e., the loss of labor market earnings and uncompensated household and caregiving functions associated with an individual's premature death from cancer—to capture the economic effects of cancer-related mortality, with the understanding that the full value of a life cannot be quantified in purely economic terms, and every individual is worth more than the value of his or her labor (14, 16–18). However, monetary estimates that translate the larger human cost of cancer into an understandable metric (i.e., dollar values) can help drive policy-making decisions, research priorities, and prevention strategies (14). The Second Panel on Cost-Effectiveness in Health and Medicine and the CDC's Advisory Committee on Immunization Practices (ACIP) recommend and accept the inclusion of productivity costs in economic analysis, such as cost-effectiveness analyses (17, 19).

Understanding the economic burden of HPV-attributable cancers, including work-related and non-work productivity loss, can help provide decision-makers in the US and other countries around the world with a more complete picture of the disease's impact on society (20, 21). The latest productivity loss data specific to HPV-associated cancers in the US were published in 2008 and based on cancer-related deaths and population data reported in 2003 by the US Cancer Statistics Working Group (22). Although the analysis found substantial mortality burden (8,302 deaths) and productivity costs due to HPV-associated cancers in 2003, it was conducted before HPV vaccination began and did not examine cancer burden attributable to HPV or report burden stratified by HPV type. Thus, an updated estimate of the economic benefits of productivity loss reduction based on these additional factors using data from 2017 can provide additional insights into the comprehensive value of 9vHPV vaccination.

The objective of this analysis was to estimate the years of potential life lost (YPLL) and productivity loss associated with an expected loss of life years due to HPV-attributable cancers in the US in 2017, stratified by cancers attributable to vaccine-preventable high-risk HPV types 16/18 and 31/33/45/52/58.

## Methods

We estimated the number of deaths due to HPV-attributable cervical, vulvar, vaginal, penile, anal, and oropharyngeal cancers in the US in the year 2017. From these data, we calculated the YPLL and total 2017 present value of future lifetime productivity (PVFLP) due to HPV-attributable cancer deaths, stratified by cancer type, HPV type, and sex.

**Figure 1:**
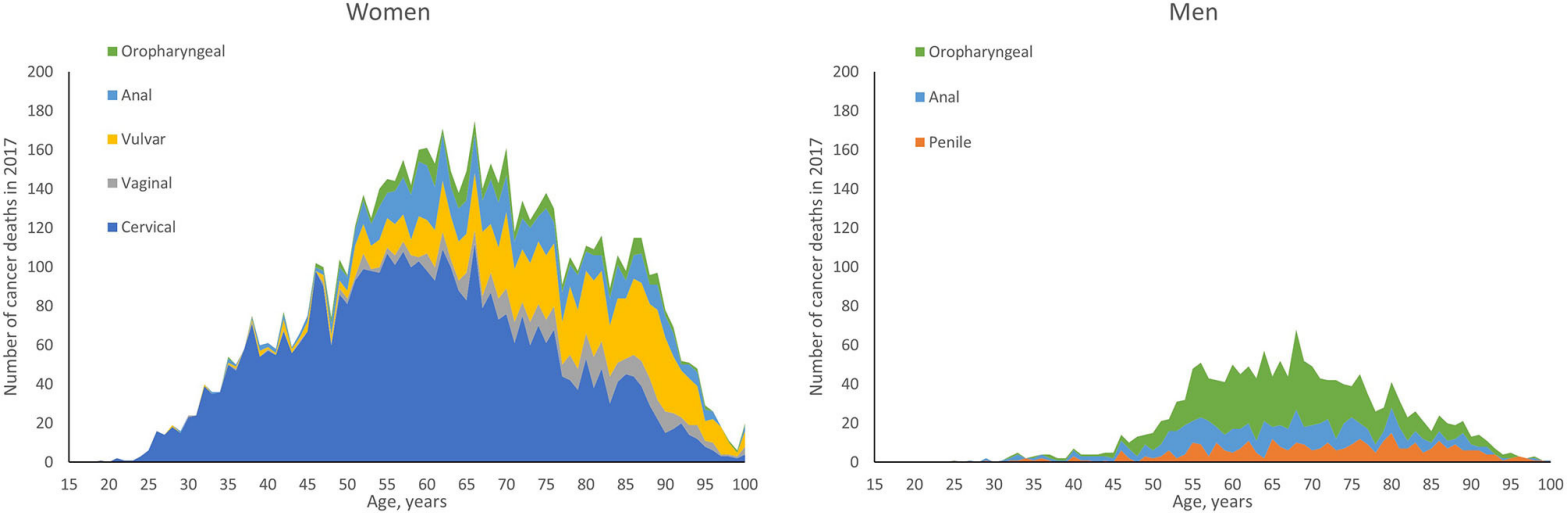
Cancer deaths by age and sex in the United States in 2017: HPV-associated cancers. HPV, human papillomavirus.

To estimate the expected number of deaths caused by HPV, the number of deaths by age and sex in each cancer site reported in the year 2017 was multiplied by either the proportion of those cancers attributable to any HPV or the proportions attributable to the high-risk HPV types targeted in the 4vHPV (HPV 16/18) or 9vHPV vaccine (HPV 31/33/45/52/58). The number of cancer-related deaths by age and sex for individuals aged 15 years and older was obtained from the CDC's Wide-ranging Online Data for Epidemiologic Research (WONDER) (23), a publicly available integrated information and communication system that provides public health data on births, deaths, cancer incidence, census data, and additional related information in the US. US mortality data from the year 2017 were used as those were the latest mortality data available at the time of our analysis in 2019. Deaths for cancers of interest were identified by *International Statistical Classification of Diseases, Tenth Revision* (*ICD-10*) codes: C53 (cervical), C51 (vulvar), C52 (vaginal), C60 (penile), C21 (anal), and C00-C14 (oropharyngeal). The WONDER-reported US cancer deaths by age and sex in the year 2017 are shown in Figure 1. The proportion of these cancer-related deaths attributable to the 4vHPV- and 9vHPV-targeted types was obtained from published CDC data from 2008 through 2010 (Table 1) (1). It is worth noting that the distribution of HPV-attributable cancer deaths may not be the same as the distribution of HPV-attributable cancer cases due to differences in disease severity and screening.

**Table 1:** Input: proportion (%) of cancers attributable to any HPV and by HPV type in the United States in 2008–2010 (1).

**Cancer site**	**Any HPV**	**HPV 16/18**	**HPV 31/33/45/52/58**
Cervix	90.6	66.2	14.7
Vagina	75.0	55.1	18.3
Vulva	68.8	48.6	14.2
Penis	63.3	47.9	9.0
Anus			
Female	92.5	79.5	10.8
Male	88.7	79.1	3.8
Oropharynx			
Female	63.3	50.8	9.5
Male	72.4	63.4	4.4

*HPV, human papillomavirus*.

YPLL was calculated for each death using the sex-specific life expectancy at the age of death taken from the US life table from 2016 (24). Specifically, the number of deaths associated with each cancer was multiplied by the percentage of that cancer attributable to HPV; the number of deaths at each age was then multiplied by the sex-specific life expectancy at the age of death. For example, 6 cervical cancer deaths were reported among 25-year-olds in 2017, and 91% of cervical cancer deaths are attributable to HPV, leading to 5.46 HPV-attributable cervical cancer deaths at age 25 (product of number of deaths and attribution proportion). The future life expectancy at age 25 for women is 57 years; thus, the total YPLL for a 25-year-old woman who died of cervical cancer is 311.22 years (product of HPV-attributable deaths and life expectancy). The total estimated YPLL in this analysis includes the sum of all 2017 deaths in persons aged 15 to 100 years.

To estimate the PVFLP for each death, the economic value of market and non-market labor for each future year of lost life was estimated and discounted to 2017 US dollars. Given an individual's sex and age at death, his or her lost market earnings were calculated as the age- and sex-specific probability of labor force participation (25) multiplied by expected annualized age- and sex-specific wages (26) and a markup factor to adjust for the additional value of fringe benefits paid by employers (1.464) (27) for each year of future life lost (Table 2). For example, the future 57-year life expectancy of the aforementioned 25-year-old woman would include 57 annual estimates of future lost earnings anticipated for each remaining year of expected life. From age 25 to 29 years, her expected labor force participation is 76.4% with $724 weekly wage, leading to estimated future lost earnings of $809.79 per year for ages 26 to 29 (product of labor force participation, weekly wage rate and markup for fringe benefits, i.e., 0.764 × $724 × 1.464). From ages 30 to 34 years, her applicable labor force participation and weekly wages are 74.5% and $724, leading to estimated future lost earnings of $789.65 per year (i.e., 0.745 × $724 × 1.464). These calculations continue up to her expected age of death (82 years). Non-market productivity was calculated similarly, summing the expected age- and sex-specific value of each year of future life lost. The annualized economic value of non-market productivity was calculated as the weekly hours of non-market work performed by the non-institutionalized US population, estimated from the 2016 American Time Use Survey (ATUS) data, multiplied by the average hourly wage rate paid to personal and service occupation workers; both inputs were obtained from published CDC data (28). The sum of these two sources of future lost productivity (i.e., market and non-market productivity) comprised the total PVFLP, and all future values were adjusted to 2017 at a discount rate of 3% as recommended by the Second Panel on Cost-Effectiveness in Health and Medicine (17).

**Table 2:** Input: age- and sex-specific labor force participation, earnings, and non-wage productivity in the United States.

**Age group, years**	**2017 labor force participation, %**		**2017 median weekly wage, $** **(** **26** **)**		**2016 annual value of non-market productivity, $** **(** **28** **)** ^****a****^	
	**Men**	**Women**	**Men**	**Women**	**Men**	**Women**
15 to 16^b^	0.0	0.0	–	–	–	–
16 to 17	23.0	26.3	459	402	8,169	14,489
18 to 19	48.2	47.2	459	402	8,169	14,489
20 to 24	74.1	68.5	570	514	8,169	14,489
25 to 29	87.5	76.4	821	724	19,097	33,087
30 to 34	90.2	74.5	821	724	19,097	33,087
35 to 39	90.9	74.3	1,062	860	25,117	35,822
40 to 44	90.5	75.7	1,062	860	25,117	35,822
45 to 49	88.3	75.8	1,103	855	19,318	25,261
50 to 54	84.6	73.2	1,103	855	19,318	25,261
55 to 59	78.0	66.2	1,098	856	16,760	23,079
60 to 64	62.4	51.0	1,098	856	16,760	23,079
65 to 69	37.3	27.9	1,016	782	17,676	22,914
70 to 74	23.7	16.2	1,016	782	17,676	22,914
75+	11.5	6.0	1,016	782	11,896	13,515

*CDC, Centers for Disease Control and Prevention; US, United States; WONDER, Wide-ranging Online Data for Epidemiologic Research*.

a *Non-market productivity inflated to 2017 US dollars*.

b *The 15–16 years age group is included in this table because the CDC WONDER database reports deaths starting at age 15 years. However, no labor force participation data were available for this age group (values were assumed to be 0)*.

## Results

This analysis estimated that a total of 7,085 HPV-attributable cancer deaths occurred in the United States in 2017; of these, 6,482 (91%) deaths were attributable to the high-risk types targeted by 9vHPV (i.e., HPV 16, 18, 31, 33, 45, 52, and 58; Table 3). Total YPLL from these deaths was estimated to be 154,954, of which life years lost in women accounted for 86%. Overall, the highest YPLL was associated with cervical cancer, with a total of 100,998 years (65% of all YPLL). Among men, the highest total YPLL was attributable to HPV-related oropharyngeal cancer (51% of all male YPLL). In women, the estimated YPLL for each death ranged from 14 for vaginal and vulvar cancer to a high of 26 for cervical cancer. In men, the highest YPLL per death was 19 for anal cancer.

**Table 3:** Estimated number of HPV-attributable cancer deaths in the United States in 2017 and estimated YPLL associated with HPV-attributable cancer stratified by sex and HPV type.

	**Total**	**Women**					**Men**		
		**Cervix**	**Vagina**	**Vulva**	**Anus**	**Oropharynx**	**Penis**	**Anus**	**Oropharynx**
**Estimated deaths^a^**									
Any HPV	**7,085**	3,812	308	868	677	182	223	388	628
High-risk HPV types targeted by 9vHPV^b^	**6,482**	3,403	302	793	661	173	200	362	588
**Estimated YPLL**									
Any HPV	**154,954**	100,998	4,405	12,247	12,548	3,249	3,377	7,223	10,905
High-risk HPV types targeted by 9vHPV^b^	**141,019**	90,185	4,311	11,179	12,249	3,095	3,036	6,751	10,212
**Estimated YPLL per death**	**22**	**26**	**14**	**14**	**19**	**18**	**15**	**19**	**17**

*9vHPV, nonavalent HPV vaccine; HPV, human papillomavirus; YPLL, years of potential life lost*.

a *HPV-attributable cancer deaths were calculated based on CDC WONDER-reported total US cancer deaths per type in 2017 (i.e., 4,207 cervical; 1,262 vulvar; 411 vaginal; 352 penile; 1,169 anal; 1,154 oropharyngeal)*.

b *HPV 16, 18, 31, 33, 45, 52, and 58*.

The total PVFLP associated with all HPV-attributable cancer deaths amounted to $4.2 billion in 2017 US dollars (Table 4). HPV-attributable cancer deaths among women accounted for 84% of the total productivity losses in 2017 ($3.6 billion), whereas deaths among men caused a loss of $655 million. The largest loss of mortality-related productivity was observed among women who died due to HPV-attributable cervical cancer, resulting in a loss of $2.8 billion. Among men, total PVFLP due to HPV-attributable cancer deaths was the highest among those who died of oropharyngeal cancer ($321 million).

**Table 4:** Estimated present value of future lifetime productivity due to HPV-attributable cancer deaths by sex and HPV type (in thousands, 2017 $).

	**PVFLP By Sex and Cancer Site (% of Total PVFLP)**								
	**Total**	**Women**					**Men**		
		**Cervix**	**Vagina**	**Vulva**	**Anus**	**Oropharynx**	**Penis**	**Anus**	**Oropharynx**
Any HPV	**4,215,447 (100)**	2,847,795 (67.6)	90,885 (2.2)	256,211 (6.1)	291,883 (6.9)	73,389 (1.7)	100,938 (2.4)	232,742 (5.5)	321,605 (7.6)
HPV 16/18	**3,203,913 (76.0)**	2,080,839 (49.4)	66,770 (1.6)	180,986 (4.3)	250,861 (6.0)	58,896 (1.4)	76,381 (1.8)	207,553 (4.9)	281,626 (6.7)
HPV 31/33/45/52/58	**626,077 (14.9)**	462,059 (11.0)	22,176 (0.5)	52,881 (1.3)	34,079 (0.8)	11,014 (0.3)	14,351 (0.3)	9,971 (0.2)	19,545 (0.5)
**PVFLP per death**	**595**	**747**	**295**	**295**	**431**	**404**	**453**	**600**	**512**

*HPV, human papillomavirus; PVFLP, present value of future lifetime productivity*.

The estimated PVFLP for cancer deaths due to HPV 16/18 and HPV 31/33/45/52/58 were $3.2 billion (76%) and $626 million (15%), respectively. The average PVFLP per death among men and women were $529,248 and $608,906, respectively.

The estimated deaths, PVFLP, and PVFLP per death for all cancers in these sites, regardless of HPV attribution, are presented in Supplementary Material.

## Discussion

This study provides estimates of the YPLL and PVFLP due to premature HPV-attributable cancer deaths in the US in the year 2017, stratified by HPV types 16/18 and 31/33/45/52/58. These estimates are based on the latest data available for the US population, labor force participation, and wages.

Previously, Ekwueme et al. (22) estimated the YPLL and productivity loss from mortality due to HPV-associated cancers in the US in the pre-vaccine era in 2003. Their study reported 181,026 YPLL and a loss of $3.7 billion from HPV-associated cancer deaths in 2003, which is consistent with our estimates of 154,954 YPLL and $4.2 billion in productivity loss from HPV-attributable cancer deaths in 2017. The difference in YPLL estimates between studies is due to the attribution of HPV-associated cancer in our calculator. We assumed that a percentage of cases in each cancer site were attributable to HPV, whereas Ekwueme et al. evaluated the burden of cancers in HPV-associated sites (i.e., all cervical, vaginal, vulvar, penile, and anal cancers and a subset of oropharyngeal cancers [*ICD* codes C01-02, C09, C14]) without regard to whether any of the cases were attributable to HPV. If we were to assume that 100% of each cancer is due to HPV, our estimated YPLL would be 182,391, on par with the Ekwueme et al. study. The difference in estimated PVFLP in our study is due to the attribution of HPV-related cancers to our calculation as well as general inflation costs between the years 2003 and 2017. Assuming 100% HPV attribution to the cancer deaths, our total PVFLP estimate would be $4.9 billion in 2017 US dollars; adjusted for inflation, this value corresponds to $3.7 billion in 2003 US dollars, which is the same as the value estimated by Ekwueme et al.

It is not surprising that our estimate of 7,085 HPV-attributable cancer deaths in 2017 is fairly similar to the Ekwueme et al. estimate of 8,302 HPV-associated cancer deaths in 2003. The ongoing HPV vaccination program is not expected to impact US cancer deaths yet because of the long lead times between HPV infection and cancer death. In a natural history study of data from women diagnosed with cervical intraepithelial neoplasia 3 (CIN3) from 1955 through 1976 in New Zealand, the median time from CIN3 diagnosis to cervical or vaginal cancer diagnosis was 27 years (29). In the US, the median age at cervical cancer diagnosis is 49 years (2), and the median age at diagnosis of high-grade cervical lesions (CIN2+) was 32 years in 2016 (30).

Although the incidence of cervical cancer attributable to 9vHPV-targeted types has decreased in the years since the Ekwueme et al. study, the incidence of head and neck cancers and other HPV-associated cancers has increased (3). Oropharyngeal cancer is now the most common cancer attributable to 9vHPV-targeted types in the US, and the rise in the total number of cancers attributable to 9vHPV-targeted types has been driven by increases in oropharyngeal, anal, and vulvar cancers among adults aged ≥40 years (3, 31). The median age at diagnosis of oropharyngeal cancer is 63 years in women and 61 years in men. Similarly, anal cancer (62 years in women, 59 years in men) and vulvar cancer (66 years) are also diagnosed later in life (2), indicating that the delay in observing population-level benefits of HPV vaccination for these cancers is likely to be longer.

Overall, HPV-attributable cancer deaths in women accounted for the highest number of YPLL in our analysis and represented 86% of the total YPLL. HPV-attributable cervical cancer deaths were associated with the highest total YPLL and YPLL per death in our analysis, contributing to the higher burden in women as compared to men. Similar results were estimated by Ekwueme et al.: HPV-associated cancer deaths in women accounted for 71% of total YPLL in their analysis, and HPV-associated cervical cancer was associated with the highest YPLL and YPLL per death (22). The high burden of HPV-attributable cervical cancer among women in our analysis may be due to the younger median age at cervical cancer diagnosis (49 years) compared to the diagnosis of other HPV-associated cancers (62–68 years) (2) and may reflect the higher labor force participation and market and non-market productivity of younger women compared to older women.

Among men, the average YPLL per death in our analysis was the highest among those who died of HPV-attributable anal cancer, and the total YPLL was the highest among those who died of HPV-attributable oropharyngeal cancer; the same rankings were observed in the Ekwueme et al. study of HPV-associated cancer deaths. The higher average YPLL per death seen with HPV-attributable anal cancer may be due to the younger median age at diagnosis (59 years) resulting in more YPLL, whereas the higher total YPLL observed with HPV-attributable oropharyngeal cancer may be attributable to the higher overall volume of oropharyngeal cancer deaths (2).

Our results show that the economic burden of HPV-attributable cancer in men is not trivial. HPV-attributable cancer deaths among men in 2017 caused a total future productivity loss of $655 million. Although the average PVFLP per HPV-attributable cancer death overall was higher among women ($608,906) than men ($529,248), the PVFLP of HPV-attributable anal and oropharyngeal cancers in our analysis was higher for men compared to women. For example, there were 19 YPLL per anal cancer death for both men and women, but the PVFLP per death was much higher for men: ~$600,000 vs. $431,000 for women. For oropharyngeal cancer, the YPLL per death is slightly lower among men than women (17 vs. 18, respectively), but again, the PVFLP is higher for men ($512,000 vs. $404,000). These differences are driven by higher labor force participation and higher wages for men compared to women.

In addition to the estimation of YPLL and PVFLP due to any HPV, we estimated these outcomes for the high-risk 9vHPV-targeted types (HPV 16/18/31/33/45/52/58). Cancer deaths caused by high-risk 9vHPV-targeted types accounted for 91% of the total YPLL and total PVFLP. These results highlight that more than three-quarters of HPV-related cancer deaths in 2017 are vaccine preventable and are associated with a significant economic burden of mortality and associated productivity loss among men and women in the US.

Our analysis was specific to the US, but the YPLL and PVFLP due to premature HPV-attributable cancer deaths could be estimated in the same manner for any country if sufficient data are available. For example, nearly 90% of cervical cancer deaths worldwide in 2018 occurred in low- and middle-income countries, and more than 60% of women with cervical cancer in these countries die from the disease, which is more than twice the proportion in high-income countries (~30%) (32). Thus, although life expectancy may be lower in low- and middle-income countries compared with high-income countries such as the US, the economic burden of premature HPV-attributable cancer deaths is still likely to be substantial in these countries. Future studies should evaluate the economic burden of premature HPV-attributable cervical cancer deaths in low- and middle-income countries where the disease burden is the greatest and inequities in HPV vaccination, screening, and treatment persist.

In August 2020, the World Health Assembly adopted a global strategy to accelerate cervical cancer elimination by focusing on prevention through HPV vaccination, screening and treatment of precancerous lesions, and treatment and palliative care for invasive cervical cancer (32). Although HPV vaccination provides primary prevention, it will take time for the benefits to appear, so increasing screening and improving access to care are key interventions to address cervical cancer burden. The World Health Organization estimated that cervical cancer elimination would add an estimated $28 billion to the world's economy by 2030, as an estimated 250,000 women would remain productive members of the workforce, resulting in gains of $700 million as a direct result of increased workforce participation and about $27 billion as an indirect benefit of good health. However, the statement also acknowledged the non-labor-market value of women who are raising children, caring for their families, and contributing to the social and economic fabric of their communities, a burden that is captured in our analysis but also needs to be evaluated in future studies. This new global strategy is a reminder that indirect costs, such as productivity losses, should be incorporated into vaccine evaluations, as recommended by the Second Panel on Cost-Effectiveness in Health and Medicine and ACIP (17, 19), to ensure that all benefits and costs associated with vaccination are captured accurately.

The results of our analysis should be interpreted with similar limitations to the study by Ekwueme et al. (22). Productivity losses are based on average wages, which may overestimate the burden if HPV-attributable cancers are not equally distributed among individuals in all socioeconomic strata, and there is evidence that individuals of lower socioeconomic status have a higher incidence of many HPV-associated cancers (33). In addition, this analysis considers the economic burden associated *after* mortality. The direct medical, non-direct medical, and productivity costs to patients and their caregivers during the course of the disease can also be substantial (34, 35) but are not quantified in our analysis. The estimates of HPV type attribution used in this analysis are based on a nationwide US cross-sectional study of 2,670 samples of archived tissue based on diagnoses from 1993 to 2005, conducted by Saraiya et al. (1). We feel that it is unlikely that attribution proportions have changed significantly between 2005 and 2017, although the possibility cannot be ruled out. Finally, as noted by Saraiya et al. (1), we acknowledge that the detection of HPV DNA in a cancer tissue does not necessarily imply causality.

Our results suggest that HPV-attributable cancer deaths are associated with a substantial economic burden in the US, with a majority of the burden due to the seven high-risk types targeted by the 9vHPV vaccine. This analysis, based on 2017 CDC data, is the first to estimate the economic burden of cancer deaths attributable to HPV (vs. deaths associated with HPV) in the US and the first conducted since the initiation of routine vaccination against HPV. Vaccination with 9vHPV may prevent HPV-attributable cancers and the associated losses (1), and our updated mortality and productivity loss estimates can inform future economic analyses and be used to help decision-makers in the US and elsewhere to assess the comprehensive value of 9vHPV vaccination.

## Data Availability Statement

Merck Sharp & Dohme Corp., a subsidiary of Merck & Co., Inc., Kenilworth, NJ, USA's data sharing policy, including restrictions, is available at http://engagezone.msd.com/ds_documentation.php. Requests for access to the study data can be submitted through the EngageZone site or via email to dataaccess@merck.com.

## Author Contributions

All authors were involved in the conception or design of the work, the acquisition, analysis, or interpretation of data, drafting the manuscript, revised and/or reviewed the manuscript for important intellectual content, provided final approval of the version to be published, and agreed to be accountable for all aspects of the work in ensuring that questions related to the accuracy or integrity of any part of the work are appropriately investigated and resolved.

## Conflict of Interest

VP, BK, CN, and DC are employees of Merck Sharp & Dohme Corp., a subsidiary of Merck & Co., Inc., Kenilworth, NJ, USA, who may own stock and/or hold stock options in Merck & Co., Inc., Kenilworth, NJ, USA. MP, SS, and SC are employees of Pharmerit – an OPEN Health Company, paid consultants to Merck Sharp & Dohme Corp., a subsidiary of Merck & Co., Inc., Kenilworth, NJ, USA. The remaining author declares that the research was conducted in the absence of any commercial or financial relationships that could be construed as a potential conflict of interest.
