# Corrigendum: Economic Value of Lost Productivity Attributable to Human Papillomavirus Cancer Mortality in the United States

**DOI:** 10.3389/fpubh.2021.691634

**Published:** 2021-07-26

**Authors:** Masoom Priyadarshini, Vimalanand S. Prabhu, Sonya J. Snedecor, Shelby Corman, Barbara J. Kuter, Chizoba Nwankwo, Diana Chirovsky, Evan Myers

**Affiliations:** ^1^Pharmerit – an OPEN Health Company, Bethesda, MD, United States; ^2^Merck & Co. Inc., Kenilworth, NJ, United States; ^3^Division of Women's Community and Population Health, Department of Obstetrics & Gynecology, Duke University School of Medicine, Durham, NC, United States

**Keywords:** human papillomavirus, productivity loss, mortality, cervical cancer, oropharyngeal cancer

In the original article, there was a calculation error underestimating the number of cancer deaths attributable to 9vHPV-targeted types.

In Table 3, the numeric values in the two rows with the subheading High-risk HPV types targeted by 9vHPV have been updated. The corrected Table 3 appears below.

**Table 3:** Estimated number of HPV-attributable cancer deaths in the United States in 2017 and estimated YPLL associated with HPV-attributable cancer stratified by sex and HPV type.

	**Total**	**Women**					**Men**		
		**Cervix**	**Vagina**	**Vulva**	**Anus**	**Oropharynx**	**Penis**	**Anus**	**Oropharynx**
**Estimated deaths^a^**									
Any HPV	**7,085**	3,812	308	868	677	182	223	388	628
High-risk HPV types targeted by 9vHPV^b^	**6,482**	3,403	302	793	661	173	200	362	588
**Estimated YPLL**									
Any HPV	**154,954**	100,998	4,405	12,247	12,548	3,249	3,377	7,223	10,905
High-risk HPV types targeted by 9vHPV^b^	**141,019**	90,185	4,311	11,179	12,249	3,095	3,036	6,751	10,212
**Estimated YPLL per death**	**22**	**26**	**14**	**14**	**19**	**18**	**15**	**19**	**17**

*9vHPV, nonavalent HPV vaccine; HPV, human papillomavirus; YPLL, years of potential life lost*.

a *HPV-attributable cancer deaths were calculated based on CDC WONDER-reported total US cancer deaths per type in 2017 (i.e., 4,207 cervical; 1,262 vulvar; 411 vaginal; 352 penile; 1,169 anal; 1,154 oropharyngeal)*.

b *HPV 16, 18, 31, 33, 45, 52, and 58*.

In Table 4, the numeric values in the two rows with the subheadings HPV 16/18 and HPV 31/33/45/52/58 have been updated. The corrected Table 4 appears below.

**Table 4:** Estimated present value of future lifetime productivity due to HPV-attributable cancer deaths by sex and HPV type (in thousands, 2017 $).

	**PVFLP By Sex and Cancer Site (% of Total PVFLP)**								
	**Total**	**Women**					**Men**		
		**Cervix**	**Vagina**	**Vulva**	**Anus**	**Oropharynx**	**Penis**	**Anus**	**Oropharynx**
Any HPV	**4,215,447 (100)**	2,847,795 (67.6)	90,885 (2.2)	256,211 (6.1)	291,883 (6.9)	73,389 (1.7)	100,938 (2.4)	232,742 (5.5)	321,605 (7.6)
HPV 16/18	**3,203,913 (76.0)**	2,080,839 (49.4)	66,770 (1.6)	180,986 (4.3)	250,861 (6.0)	58,896 (1.4)	76,381 (1.8)	207,553 (4.9)	281,626 (6.7)
HPV 31/33/45/52/58	**626,077 (14.9)**	462,059 (11.0)	22,176 (0.5)	52,881 (1.3)	34,079 (0.8)	11,014 (0.3)	14,351 (0.3)	9,971 (0.2)	19,545 (0.5)
**PVFLP per death**	**595**	**747**	**295**	**295**	**431**	**404**	**453**	**600**	**512**

*HPV, human papillomavirus; PVFLP, present value of future lifetime productivity*.

A correction has been made to some numeric values within the abstract, results, and discussion:

**Abstract Results:** “An estimated 7,085 HPV-attributable cancer deaths occurred in 2017 accounting for 154,954 YPLL, with **6,482** deaths (**91**%) and **141,019** YPLL (**91**%) attributable to 9vHPV-targeted types. The estimated PVFLP was $**3.8** billion for cancer deaths attributable to 9vHPV-targeted types (84% from women). The highest productivity burden was associated with cervical cancer in women and anal and oropharyngeal cancers in men.”

**Results sentence 1**: “This analysis estimated that a total of 7,085 HPV-attributable cancer deaths occurred in the United States in 2017; of these, **6,482** (**91**%) deaths were attributable to the high-risk types targeted by 9vHPV (i.e., HPV 16, 18, 31, 33, 45, 52, and 58; Table 3).”

**Results paragraph 3**: “The estimated PVFLP for cancer deaths due to HPV 16/18 and HPV 31/33/45/52/58 were $**3**.2 billion (**76**%) and $**626** million (**15**%), respectively. The average PVFLP per death among men and women were $529,248 and $608,906, respectively.”

**Discussion paragraph 8**: “Cancer deaths caused by high-risk 9vHPV-targeted types accounted for **91**% of the total YPLL and total PVFLP.”

In the original article, there was a mistake in the discussion that has been corrected:

**Discussion paragraph 10**: “However, the statement also acknowledged the non-labor-market value of women who are raising children, caring for their families, and contributing to the social and economic fabric of their communities, a burden that **is** captured in our analysis **but** also needs to be evaluated in future studies.”

The authors apologize for the errors and state that this does not change the scientific conclusions of the article in any way. The original article has been updated.

## Publisher's Note

All claims expressed in this article are solely those of the authors and do not necessarily represent those of their affiliated organizations, or those of the publisher, the editors and the reviewers. Any product that may be evaluated in this article, or claim that may be made by its manufacturer, is not guaranteed or endorsed by the publisher.

