# Supplementary material for: Economic Value of Lost Productivity Attributable to Human Papillomavirus Cancer Mortality in the United States
Source: Front Public Health. 2021 Feb 16;8:624092. doi: 10.3389/fpubh.2020.624092 (PMC7921151; doi:10.3389/fpubh.2020.624092)
Supplement: Supplementary file 1 [file Data_Sheet_1.docx]

**Estimated deaths, PVFLP, and PVFLP per death for cancers, regardless of HPV attribution**

The estimated deaths, present value of future lifetime productivity (PVFLP), and PVFLP per death for all cancers in sites where human papillomavirus (HPV) is often found, regardless of HPV attribution, are presented in Supplemental Table 1. An estimated 8,555 cancer deaths occurred in these sites in 2017 in the United States, with deaths in women accounting for 81% of deaths. The highest number of deaths was associated with cervical cancer (n=4,207; 49% of total deaths, 61% of female deaths). Among men, oropharyngeal cancer was associated with the highest number of deaths (n=867; 10% of total deaths, 52% of male deaths). The total PVFLP associated with these deaths was $4.9 billion, with cancer deaths among women accounting for 82% ($4.0 billion). The largest mortality-related productivity loss was observed among women who died due to cervical cancer ($3.1 billion); among men, the largest loss was associated with oropharyngeal cancer ($444 million). The estimated PVFLP per death was $576,784; values ranged from $294,843 (vaginal cancer) to $747,150 (cervical cancer) in women and from $453,009 (penile cancer) to $600,441 (anal cancer) in men.

**Supplemental Table 1** Estimated present value of future lifetime productivity due to cancer deaths by sex and cancer site

|  | **Total** | **Women** | | | | | **Men** | | |
| --- | --- | --- | --- | --- | --- | --- | --- | --- | --- |
|  |  | **Cervix** | **Vagina** | **Vulva** | **Anus** | **Oropharynx** | **Penis** | **Anus** | **Oropharynx** |
| **Estimated deaths** | **8,555** | 4,207 | 411 | 1,262 | 732 | 287 | 352 | 437 | 867 |
| **Estimated PVFLP^a^** | **4,934,385** | 3,143,261 | 121,180 | 372,400 | 315,549 | 115,938 | 159,459 | 262,393 | 444,205 |
| **Estimated PVFLP per death^a^** | **577** | 747 | 295 | 295 | 431 | 404 | 453 | 600 | 512 |

Abbreviations: PVFLP, present value of future lifetime productivity.

^a^ In thousands, 2017 United States dollars ($)
